# Dementia Specialization Among Adult Day Services Centers: National Study of Long-Term Care Providers

**DOI:** 10.1093/geroni/igab046.320

**Published:** 2021-12-17

**Authors:** Jessica Lendon, Manisha Sengupta, Amanuel Melekin

**Affiliations:** 1 National Center for Health Statistics, Hyattsville, Maryland, United States; 2 CDC/NCHS, Hyattsville, Maryland, United States

## Abstract

Adult day services centers (ADSC) are a source of community-based care for persons with Alzheimer’s disease/other dementias. This study compares dementia specialized ADSCs (DSADSC) and their participants to other ADSCs that do not specialize in dementia care using the 2016-2018 National Study of Long-term Care Providers. DSADSCs account for 10% of all ADSCs and serve 15% of all ADSC participants with dementia. About half of DSADSC participants have dementia, compared to 30% in other ADSCs. A higher percentage of DSADSCs, compared to other ADSCs, were in the Midwest, were nonprofit, had a social model, and employed nursing aides. Fewer DSADSCs, compared to other ADSCs, provided nursing, mental health, and transportation services. More DSADSC participants were 75 years of age or older and needed assistance with eating and toileting. Findings may help identify how ADSCs, particularly, DSADSCs, meet the unique care needs of older adults with dementia.

